# The Prevalence of Responders and Non-Responders for Body Composition, Resting Blood Pressure, Musculoskeletal, and Cardiorespiratory Fitness after Ten Weeks of School-Based High-Intensity Interval Training in Adolescents

**DOI:** 10.3390/jcm12134204

**Published:** 2023-06-21

**Authors:** Jarosław Domaradzki, Dawid Koźlenia, Marek Popowczak

**Affiliations:** 1 Unit of Biostructure, Faculty of Physical Education and Sport, Wroclaw University of Health and Sport Sciences, al. I.J. Paderewskiego 35, 51-612 Wroclaw, Poland; jaroslaw.domaradzki@awf.wroc.pl; 2 Unit of Team Sports Games, Faculty of Physical Education and Sport, Wroclaw University of Health and Sport Sciences, al. I.J. Paderewskiego 35, 51-612 Wroclaw, Poland; marek.popowczak@awf.wroc.pl

**Keywords:** HIIT, physical education, interindividual variability, biological maturation, resting blood pressure, cardiorespiratory fitness, musculoskeletal fitness, body mass composition

## Abstract

Many observations have demonstrated great heterogeneity in the magnitude of the response to the physical exercise stimulus. Only a few studies have investigated the effects of high-intensity interval training (HIIT) on the prevalence of non-responders (NRs) in adolescents while considering health-related fitness measurements and other co-variables. Therefore, the aim of the current work was twofold: (1) to assess the effects of ten weeks of school-based HIIT on the prevalence of responders (Rs) and NRs for body composition, resting blood pressure (BP), cardiorespiratory and musculoskeletal fitness, and (2) to assess the relationship of this prevalence with sex, body mass category, and biological maturation status, while estimating the likelihood of a response. Adolescents aged 16 years (*n* = 73) were included in the study. Waist-to-hip ratio, body fat percentage (BFP), skeletal muscle mass (SMM), BP, cardiorespiratory fitness (CRF), and musculoskeletal fitness were the primary outcomes. Co-variables included sex, body mass index (BMI), and maturity offset. The percentages of the Rs and NRs were analyzed based on changes (Δ) between post-intervention and pre-intervention values. The typical error (TE) method was used to identify Rs and NRs. Results showed a variation in the relative size of changes (% of changes) depending on the variable and sex. The greatest changes were observed in females’ abdomen muscle strength (Δ% = 23.89%), and the difference from that of males (Δ% = 5.98%) was statistically significant (*p* < 0.001) with very large effect size of (Cohen’s d = 0.941). Similar significant differences in relative changes were observed in body composition, but in the reverse direction. Males gained more from HIIT in the decrease in the body fat percentage (Δ% = −8.24%) and increase in skeletal muscle mass (Δ% = 3.38%) comparing to females (Δ% = 0.46, *p* = 0.040 and Δ% = 1.06%, *p* = 0.007, respectively). Effect size for body fat was small (Cohen’s d = 0.469), and for skeletal muscle mass it was moderate (Cohen’s d = −0.659). The results also showed positive significant differences in the prevalence of Rs compared to NRs in BFP, SMM, systolic BP (SBP), diastolic BP (DBP), CRF, and abdominal muscular strength (ABS) (*p* = 0.047, *p* = 0.047, *p* < 0.001, *p* = 0.003, *p* = 0.014, and *p* = 0.014, respectively). The effect was greatest and largest for SBP (Cohen’s ω = 0.67). Sex was related to the prevalence of Rs for ABS and close to significance for SMM. More males than females benefited from HIIT in SMM (*p* = 0.058), but more females than males had ABS benefits (*p* = 0.050). Males were more likely (2.5-fold) to be Rs than females in SMM. BMI was not related to the prevalence of Rs and NRs. Biological maturation was related to the prevalence of Rs and NRs in SMM (*p* = 0.036) and SBP (*p* = 0.016). In SBP, 100% of the early matured derived benefit from HIIT, but the effect size was small (Cramer’s V = 0.27). Those who maturated later were more likely (almost 70%) to be Rs. Thus, the HIIT program introduced to a typical physical education (PE) lesson can be considered partially effective. Therefore, there is a need to continue the search for an optimal and effective program for all health-related parameters. The close relationship between analyzed values and sex and biological maturation, but not BMI, indicates that the intervention programs should be tailored specifically for males and females.

## 1. Introduction

The prevalence of obesity, high blood pressure (BP), low cardiorespiratory fitness (CRF), and motor performance is a continuously growing global problem. Moreover, the relationship between all these morphological and functional areas is well known, as is their association with health [1,2,3,4,5]. Such intensity in the increase in these adverse phenomena is associated with, among others, decreased physical activity (PA) in children and adolescents [6]. Globally, over 80% of young people do not perform the minimum PA level recommended by the World Health Organization (WHO) [7], and the issue is of concern in Poland [8].

Many activities increase PA and its intensity, with the effectiveness of exercise intervention programs included in physical education (PE) lessons being studied, among others. The most common is school-based high-intensity interval training (HIIT) intervention [9,10,11,12]. Evidence from studies generally confirm that implementing HIIT into PE lessons is a useful prophylactic strategy for treating and preventing excessive body weight (particularly body fat mass), elevated BP, and improving muscle strength, cardiovascular function, and functional capacity [13,14,15,16]. Given the increasing popularity of HIIT in Europe, it would be beneficial to consider incorporating this type of intervention into PE lessons [17]. Although HIIT is not as widely recognized globally as it is in Europe, researching this intervention may contribute to its broader acceptance and popularity [18].

Despite positive results commonly shown for mean values of intervention-related changes in whole participant groups, there is a wide interindividual variation in the results obtained [19,20,21]. Even when the stimulus is consistent (type, strength, direction, etc.), some individuals benefit from intervention, usually called “responders” (Rs), and some have no or worsened responses, termed “non-responders” (NRs) [22,23,24,25]. The phenomenon of interindividual variability in response to exercise training (IVRET) indicates that, although the mean value of the group suggests significant improvement in variables (e.g., decrease in body fat mass) after training, there are individuals in this intervention group who show no changes. 

Studies on Rs and NRs are usually based on different methodological procedures, with variations in calculating measurements and cutoff values for classifying participants and identifying Rs and NRs. A short review of the most common methods was presented by Dankel and Loenneke [26]. Regardless of the methods used, most studies were related to training effects (resistance, power, or interval training) in adults, clinical patients, or athletes [21,27,28,29], with fewer studies based on HIIT. Moreover, there is a lack of studies on children and young people based on HIIT implemented in PE lessons. Furthermore, to the best of our knowledge, there are few studies on the prevalence of Rs and NRs to a range of dependent variables defining health-related fitness (H-RF), including sex, body mass index (BMI), and biological maturation. The only study with such a design addressed insulin-resistant school children [30].

The present study assessed the effects of a ten-week school-based HIIT program on the prevalence of Rs and NRs for body composition, resting BP, CRF, and musculoskeletal fitness. A second aim was to assess the relationship of this prevalence with sex, BMI, and biological maturation status, together with an estimation of the likelihood of a response to the exercise stimuli. It was hypothesized that: (1) there would be various effects of HIIT in R and NR frequencies and different morphological and functional variables; and (2) independent of the intervention-induced changes, there would be similar effects of HIIT on the prevalence of Rs and NRs between categories in the highlighted groups (sex, BMI, and maturity offset (MO)). 

## 2. Materials and Methods

The current work is part of the project “Physical activity and nutritional education in preventing civilization diseases-theoretical aspects and practical implications for the secondary school physical education program”, and was carried out in secondary schools in Wroclaw, a city in the Lower Silesia region of Poland. Detailed information about participants, procedures, examinations, main statistical characteristics (pre- and post-intervention), and percentages of participants in relation to BMI categories and body fat, etc., with statistical significance of the comparisons between experimental and control groups is presented elsewhere [11,31]. Here, the most critical and basic information is presented. 

Before running the project, the G*Power (version 3.1.) was used to calculate the a pri-ori sample size. Bearing in mind the main statistical analysis conducted to study the effects of the HIIT on outcomes in experimental group (EG) and control group (CG) separated into male and females (mixed effect analysis of variance (ANOVA), with within-subject and between-subjects effects), the effect size of 0.25 (medium effect size), a *p*-value of 0.05, power of 0.80, number of groups (4), and two measurements, the suggested total sample size was 179 participants [30,32]. At the beginning, 187 adolescents from secondary school in Wroclaw, Poland (66 boys: age 16.24 ± 0.34 years; 121 girls: age 16.12 ± 0.42 years) were recruited. Next, participants were randomly assigned to the experimental (EG) and control (CG) groups. Finally, 141 participants completed the study, comprising 52 boys (EG N = 31; CG N = 21; age, 16.24 (±0.34) years; body height, 176.74 (±6.07) cm; body mass, 65.42 (±12.51) kg) and 89 girls (EG N = 42; CG N = 47; age, 16.12 (±0.42) years; body height 164.38 (±6.54) cm; body mass 56.71 (±10.23) kg).

### 2.1. Participants

**Figure 1:**
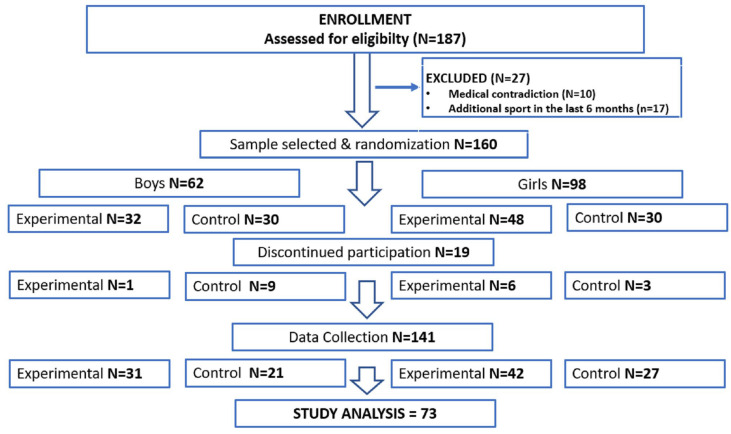
Flow-chart diagram. The flow of participants through the study.

In this work, only experimental groups (N = 73) were analyzed due to the aim focused on the effectiveness of the HIIT intervention in changes in morphological, physiological, and motor performance variables. Effectiveness was assessed through number of the responders to the exercise stimulus. The flow of the participants in this study, with the final numbers of participants in the current work, is presented on Figure 1.

### 2.2. Procedures

Measurements were taken before and after the ten-week intervention over one day between 08:00 and 13:00. Participants were asked to excrete, avoid PA and excessive drinking of liquids, and keep their typical morning patterns directly before measurement.

### 2.3. Measurements

#### 2.3.1. Anthropometry

Body height was measured with an accuracy of 0.1 cm using an anthropometer (GPM Anthropological Instruments, DKSH Ltd., Zurich, Switzerland). Body weight and body mass composition (percentage body fat (BFP) and skeletal muscle mass (SMM)) were measured using an InBody230 body composition analyzer (In-Body Co., Ltd., Cerritos, CA, USA). This tool is characterized by very high reliability, as indicated by a high intraclass correlation coefficient (ICC) for BFP (≥0.98), FM (≥0.98), and FFM (≥0.99), and low standard error of measurements. BMI was calculated as a ratio of body weight (kg) to body height (cm^2^). Waist-to-hip ratio (WHR) was calculated as a ratio of waist circumference to hip circumference. Based on BMI, each participant was classified as lower-weighted or higher-weighted. As in previous works, a BMI = 20 was the cutoff point. 

#### 2.3.2. Resting Blood Pressure

An Omron BP710 automatic BP monitor measured BP three times after the subjects sat quietly for ten minutes, with ten-minute intervals between measurements. Analysis used the means of the three measurements. Systolic BP (SBP) and diastolic BP (DBP) were noted.

#### 2.3.3. Cardiorespiratory Fitness-Fitness Index (FI)

The Harvard Step Test (HST) evaluated aerobic capacity, with the results used to calculate the fitness index (FI) according to the following formula [33]: FI = (100 × L)/(5.5 × p), where L = duration of the test in seconds, L < 300 s, and p = heart rate (HR) within 90 s of the subject stopping the test. The reliability of the HST was acceptable, with an ICC of 0.63 [34].

#### 2.3.4. Musculoskeletal Fitness (MSF)

Hand muscular strength (HS), 30-s sit-up test (ABS), and sit-and-reach test (flexibility—FLEX) were assessed. All tests were performed according to the Eurofit guidelines [35], with the Eurofit tests demonstrating very good test–retest reliability and validity [36]. The 4 × 10 m shuttle run test (agility—AG) and the vertical jump (VJ) test (leg power) determined the motor component. All measurements met the HR-F guidelines. The scientific rationale for selecting all the tests used, including their reliability for young people, has been previously verified [5,33].

### 2.4. Intervention

A PE lesson with a total duration of 45 min started with a standardized ten-minute warm-up, including five minutes of slow jogging and five minutes of stretching (dynamic and static). The main activity was 14 min of TAP, which comprised three sessions of four minutes. The Tabata protocol for each session consisted of eight cycles of two exercises, with the first session involving push-ups and high knees, the second session including dynamic lunges and spider crawl, and the third implementing a plank to push-up and side squeeze [37,38,39]. Each cycle started with as many repetitions as possible of a maximum-intensity exercise lasting for 20 s, then 10 s of active rest. To verify exercise intensity during the TAP, adolescents’ maximum HR (HRmax) was determined using the formula HRmax = 208 – 0.7 × age (16 years) [40]. The HRmax (197 bpm) was used to compute the high-intensity exercise ranging from 75% to 80% of HRmax (145–157 bpm). Students’ heart rates were monitored during the first PE lesson using a Polar H1 HR monitor (Polar Electro, Kempele, Finland). The monitors were fitted to each student’s chest level with the xiphoid process and underneath their clothing. HR was displayed on the Polar H1 watch screens during TAP exercises to encourage users to maintain adequate intensity. The EG achieved a mean HR = 155.8 bpm (±18.2; 95% confidence intervals (CIs) = 121–184). In subsequent lessons, the intensity of HR measurement was similar to that during the first PE lesson.

### 2.5. Prediction of Age at Peak Height Velocity (APHV)

Variation in the tempo of biological development was assessed based on indicators of the moment of maturity for both sexes in the EG and CG using formulas proposed by Moore et al. [41]. Age at peak height velocity (APHV) was predicted using sex-specific regression equations:

females MO = −7.709133 + (0.0042232 × (age × BH));

males MO = −7.999994 + (0.0036124 × (age × BH)).

APHV was calculated as calendar age − MO for all individuals.

Each participant was classified as a biologically younger adolescent (BYA) or biologically advanced adolescent (BAA). BYAs maturated later (so their MO was lower), while BAAs maturated earlier and had a higher MO. 

Classification to BYA and BAA was based on a regression strategy, with MO regressed on calendar age. Two regressions were considered, including simple and polynomial 2nd term. The criterion for the choice was the determinant coefficient (R^2^). Models with higher R^2^ were considered when searching for a classification cutoff point for earlier and later maturated individuals. Results confirmed better fitting polynomial curves for males (simple regression R^2^ = 0.25 vs. squared regression R^2^ = 0.29) and females (simple regression R^2^ = 0.34 vs. squared regression R^2^ = 0.40). Polynomial models were statistically significant for both sexes.

**Figure 2:**
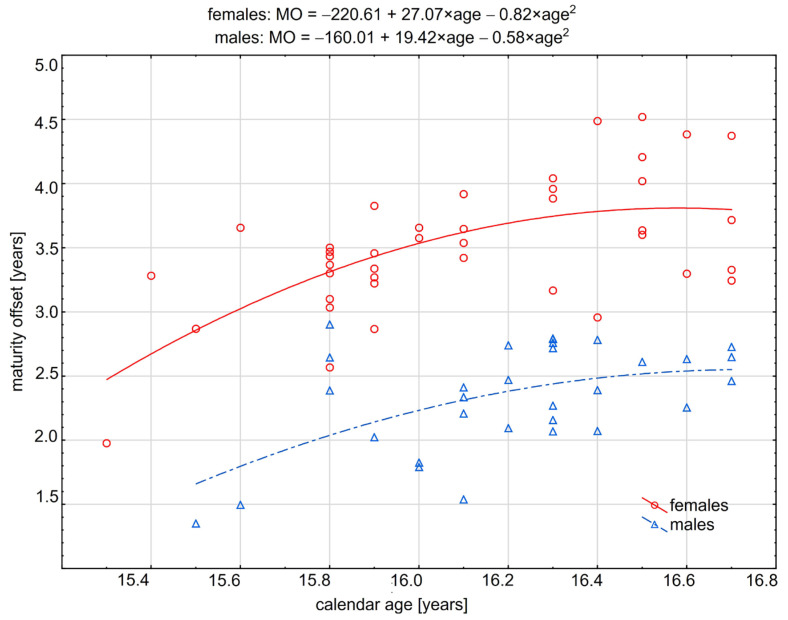
Squared functions with regression equations for males and females.

Next, the maximum value of the quadratic function was computed. Using the equation y = ax^2^ + bx + c, the following formula calculated the peak value: max = c − (b^2^/4a). The results were plotted and presented as scatterplots (Figure 2).

Finally, cutoff points for MO were 2.57 for males and 3.92 for females.

### 2.6. Classification of Responders (Rs) and Non-Responders (NRs)

Rs and NRs were defined as individuals who did or did not experience benefits following the completion of the exercise training intervention [42,43]. To classify the participants as Rs and RNs, BFP, BP, AG, SMM, FI, HS, ABS, FLEX, VJ, and TE were calculated similarly to recent studies [20,30]. The following equation was used:

$$TE=SD_{diff}/\sqrt{2}$$

where *TE* is the typical error and *SD_diff_* is the standard deviation of the difference (change) between the post-intervention and pre-intervention values.

Rs were classified as participants who demonstrated a decreased or increased difference (depending on beneficial changes) greater than a 2-fold TE away from zero [20]. 

Thus, the cutoffs values, separately for males (M) and females (F), were the following: WHR: M = 0.048 and F = 0.032; BFP: M = 6.298 and F = 3.245; SMM: M = 1.057 and F = 1.405; SBP: M = 12.407 and F = 11.634; DBP: M = 10.546 and F = 12.920; FI: M = 5.968 and F = 5.710; HS: M = 3.964 and F = 3.204; ABS: M = 5.357 and F = 4.711; FLEX: M = 4.354 and F = 4.100; AG: M = 0.761 and F = 0.801; VJ: M = 8.584 and F = 8.145. 

### 2.7. Statistics

Data are presented as mean, SD, and 95% CIs. Shapiro–Wilk’s, Levene’s, and Mauchly’s tests with Greenhouse–Geisser correction assessed data normality, homoscedasticity, and sphericity, respectively. Delta values (Δ) were calculated by subtracting pre-intervention values from post-intervention values. To test sex-specific differences in Δ, Student’s *t*-test for independent groups was conducted. Meanwhile, a chi-squared goodness-of-fit test compared the prevalence of Rs and NRs for all variables to test whether the observed frequencies were significantly different from what was expected, such as equal frequencies. To determine differences between categorical variables for Rs and NRs by covariate variable categories (sex, MO, and BMI), a chi-squared test of independence was used. Odds ratios (OR) of NR status to HIIT by groups (sex, BMI, MO) were calculated. An OR > 2 indicated a high risk of being an NR. Cohen’s ω and Cramer’s V statistics were calculated to compare ES between variables [44]. Classification of the ES was as follows: trivial (<0.1), small (0.1–0.3), medium (0.3–0.5), or large (>0.5).

The alpha level was fixed a *p* < 0.05 for all tests of statistical significance. All calculations (except Cohen’s ω) employed Statistica 13.0 software (StatSoft Poland, Krakow, Poland). Cohen’s ω was calculated in R software (R version 4.2.2) with RStudio v. 2023.03.0 Build 386 (PBC, Boston, MA, USA, URL http://www.rstudio.com/ (accessed on 15 May 2023)) using the rcompanion package v. 2.4.30 [45].

## 3. Results

The primary outcomes of this study were Δ for the indicated variables in the experimental groups. Baseline and post-intervention values, and all detailed comparisons, relationships, and exploratory analyses, were presented in previous works [11,14,31]. Here, assuming that EG males and EG females did not differ from those at the baseline (except for sex-specific differences), the groups had similar initial levels of body composition and aerobic and motor performance. The primary analyses showed a statistically significant HIIT effect on body fat mass, FI, SBP, and (dependent on sex) ABS and FLEX (*p* < 0.05).

The somatic characteristics of the EG males (*n* = 31 (16.20 ± 0.31 years) were as follows: body height—176.47 ± 6.21 cm (95% CIs = 174.19–178.74), body weight—65.24 ± 13.67 kg (95% CIs = 60.23–70.26), and BMI—20.90 ± 3.92 (95% CIs = 19.46–22.33). The main somatic characteristics of the EG females (16.12 ± 0.39 years) were as follows: body height—164.89 ± 6.08 cm (95% CIs = 162.99–166.79), body weight—56.07 ± 7.48 kg (95% CIs = 53.75–58.41), and BMI—20.57 ± 1.92 (95% CIs = 19.97–21.17).

Table 1 presents the basic statistical characteristics (mean, SD, and 95% CIs) for the ten-week HIIT-induced Δ in WHR, body mass composition, cardiovascular parameters (SBP and DBP), FI, and musculoskeletal fitness (HS, ABS, FLEX, AG, and VJ). A *t*-test for independent groups was conducted to test sex-specific differences in Δ and relative changes (Δ%). The results showed a variation in the relative size of changes (% of changes) depending on the variable and sex. The greatest changes was observed in females’ abdomen muscle strength (Δ% = 23.89%), and the difference from that of males (Δ% = 5.98%) was statistically significant (*p* < 0.001) with very large effect size of (Cohen’s d = 0.941). Similar significant differences in relative changes were observed in body composition, but in the reverse direction. Males gained more from HIIT in the decrease in body fat percentage (Δ% = −8.24%) and increase in skeletal muscle mass (Δ% = 3.38%) comparing to females (Δ% = 0.46, *p* = 0.040 and Δ% = 1.06%, *p* = 0.007, respectively). Effect size for body fat was small (Cohen’s d = 0.469), and for skeletal muscle mass it was moderate (Cohen’s d = −0.659). 

The detailed numbers and percentages of the Rs and NRs for all variables and statistical differences in frequencies between groups are presented in Table 2. There were no significant disparities in NR and R frequencies for WHR (χ^2^ = 0.34, *p* = 0.558), FLEX (χ^2^ = 2.32, *p* = 0.130), and AG (χ^2^ = 1.11, *p* = 0.292). However, there were significantly more NRs for HS (χ^2^ = 8.56, *p* = 0.003) and VJ (χ^2^ = 7.25, *p* = 0.007). As such, there was no HIIT effect in relation to common phenomena, independent of sex, though there were significant differences in BFP (χ^2^ = 3.93, *p* = 0.047), SMM (χ^2^ = 3.93, *p* = 0.047), SBP (χ^2^ = 32.89, *p* < 0.001), DBP (χ^2^ = 8.56, *p* = 0.003), FI (χ^2^ = 6.04, *p* = 0.014), and ABS (χ^2^ = 6.04, *p* = 0.014) (Table 2). Thus, the results showed that the HIIT intervention was more effective for body composition (reducing body fat and increasing SMM), cardiovascular parameters (decreasing resting BP), CRF (improving physical efficiency), and abdomen muscle strength. The greatest effect of HIIT was noted for SBP, which had a large ES (ω = 0.67). A medium ES was observed for DBP (ω = 0.34) and FI (ω = 0.29). Meanwhile, a low but noticeable ES was observed for BFP and SMM (both ω = 0.23) (Table 2).

When considering sex, BMI, and APHV as co-variables when assessing the prevalence of NRs and Rs in relation to lower and higher weighted and earlier and later maturated, significant and non-significant differences were observed for specific morphological and functional variables. The results of the chi-squared test of independence are presented in Table 3, Table 4 and Table 5. The BMI cutoff value was 20.01, and MO was 2.57 in males and 3.92 in females.

Applying sex as a factor led to the proportions of Rs and NRs mirroring the trends presented above for the whole group in both sexes. However, there were no significant differences (*p* > 0.05) in the prevalence of Rs and NRs between males and females for most variables. The only two variables that demonstrated sex-specific differences were ABS (χ^2^ = 3.83, *p* = 0.050) and SMM, where the difference was very close to significance (χ^2^ = 3.59, *p* = 0.058) (Table 3). For SMM, more Rs than NRs were noted in males, while more Rs than NRs were observed in females for ABS. The likelihood of males being Rs compared to females was 2.5-fold higher, while the females had a 60% more chance of being Rs for ABS than the males. The ES was small for SMM (V = 0.21) and ABS (V = 0.22) (Table 3). 

When using BMI as a factor, the trend in the frequencies of Rs and NRs was similar in both categories (lower BMI and higher BMI) to the whole group. More NRs than Rs were observed for HS and VJ. However, BMI categories did not affect the prevalence of the Rs and NRs, so there were no differences in the proportions of the Rs and NRs between categories (Table 4). 

Figure 2 presents the quadratic function curves with peak MO values for males and females. Parabolic, inverted L-shaped curves for both sexes illustrated constantly increasing biological maturation at the beginning of the calendar age, with a clear peak that can be considered a threshold from which MO had a constant plateau beyond a maximum. 

The trend in the frequencies of Rs and NRs was also similar to the trend in the whole group in both MO categories (BYA and BAA). More NRs than Rs were observed for HS, AG, and VJ, though there were no differences in the proportions of Rs and NRs between categories, except for SMM (χ^2^ = 4.40, *p* = 0.036) and SBP (χ^2^ = 5.78, *p* = 0.016) (Table 5). The prevalence of Rs was significantly greater for SMM in the BYA participants than in the BAA individuals. Furthermore, there were no NRs among the BAA group for SBP, with all individuals benefiting from HIIT. The chance of being an SMM R in the BYA group was almost 70% higher than in the BAA category. However, the ES for both variables was small (V = 0.24 and V = 0.27, respectively).

## 4. Discussion

The aim of this work was two-fold: (1) to assess the effects of a ten-week school-based HIIT on the prevalence of the Rs and NRs for body composition, resting BP, and cardiorespiratory and musculoskeletal fitness, and (2) to assess the relationship of this prevalence with sex, body mass category, and biological maturation status. The results indicate significant differences in the prevalence of the Rs and NRs, with more BFP, SMM, SBP, DBP, FI, and ABS Rs. There were also significant contrasting results indicating more HS and VJ NRs. Meanwhile, there were no differences in WHR, FLEX, or AG. Sex mainly differentiated ABS, with females 70% more likely to gain from HIIT intervention, while SMM results were very close to significance. In contrast, males were 2.5-fold more likely to gain positive results from HIIT. The BMI categories were not related to R and NR frequencies. Earlier maturated adolescents (biologically advanced) were more likely to have decreased SBP (100% individuals were Rs), while the later maturated (biologically younger) were more likely to gain more SMM. 

Our results confirm the effectiveness of HIIT in H-RF improvement [46]. It is well known that participation in this form of PA can improve CRF [47], body composition [48], and physical performance [49]. In recent years, various forms of HIIT have been implemented in adolescents [10] during PE lessons, which are a natural setting for PA at this age and resulted in positive outcomes [50]. Nonetheless, the prevalence of Rs and the factors affecting it required deeper investigation. Indeed, most studies focus on analyzing the general effects of HIIT, which is insufficient due to the presence of NRs [19]. Our studies showed more Tabata Rs, and the differences are significant, but there are also NRs. Therefore, it is necessary to identify why some individuals are NRs, and some are Rs. 

A visible improvement in BP was achieved, indicating that HIIT may be effectively implemented as a rehabilitation and prevention strategy for individuals with cardiovascular system issues [51]. HIIT also improved BP parameters among adolescents, particularly among hypertensive individuals [11]. In body morphology, skeletal muscle tissue increased more frequently with decreasing body fat, which is a major advantage of this intervention and was shown previously [52]. Increased body fat is one of the most significant factors in metabolic disorders [53], particularly in adolescents. Indeed, obesity is growing in this group, and it is important to address this issue using an appropriate form of PA, with HIIT appearing to be suitable [54]. Other studies introduced less effective physical fitness development programs and found a high frequency of NRs [55,56]. The development of motor skills is possible through HIIT implementation. However, such studies have varied in duration and type of HIIT, which may cause the observed differences. As such, the appropriate setting and increased workout frequency and program duration are required [47,49].

Deeper consideration of the factors differentiating the observed frequency differences indicated sex as one of the most important. Sex differences are vital, especially in adolescents, when differences between males and females become more significant [57]. Our results showed that females were 70% more likely to gain from HIIT intervention, while SMM results were very close to significance. However, a global approach demonstrated that males were 2.5-fold more likely to achieve positive results from HIIT, similar to previous studies showing differences between sexes [58]. These findings indicate that the HIIT program needs to be tailored according to sex to achieve optimal effectiveness. 

The sex difference in the integrative response to acute exercise appears to be influenced by the capacity to transport and utilize oxygen and variations in the fatigue resistance of contractile elements. [59]. However, this observation needs further investigation in adolescents, particularly in terms of biological maturation, which is another factor impacting HIIT R prevalence. In the current study, biologically older participants improved their BP parameters more frequently than their younger peers, which may be tied to BP abnormalities being more frequent among the biologically older [60]. The opposite effect was observed for muscle tissue development, with body morphology indicating that younger participants had not developed muscle mass and were more responsive to the HIIT program, which included resistance exercises. Indeed, muscle hypertrophy strongly depends on biological maturation [61]. 

The last factor considered was BMI, for which Cole et al. [62] indicated norms in adolescents and is considered useful in the early assessment of body morphology. An elevated BMI is associated with a higher risk of cardiometabolic disease [63]. However, the current study did not show a clear impact of BMI on HIIT R prevalence. Nonetheless, some studies indicated that BMI could have an influence when considering body morphology or BP parameters. 

A strength of this study was the inclusion of a wide range of outcomes covering almost all H-RF components (except the metabolic component), including morphological, motor, musculoskeletal, and CRF. We also reported the ES and OR values of NRs for each outcome. In addition, we assessed MO as a potential moderator in the response categories. The study has limitations that need to be addressed. Firstly, the study excluded prepubertal and peripubertal groups. While the age homogeneity of the studied groups is acknowledged, it is important to consider that adolescents who have already completed their pubertal period may have an advantage. This is crucial because sexual maturation can significantly impact metabolic outcomes, and accounting for the influence of puberty on metabolism is essential for ensuring the validity of the findings [21]. To mitigate this limitation, we controlled for sexual maturation by implementing measures such as calculating MO. Another limitation was the absence of nutritional aspects in the investigation and the effectiveness of interval protocols used in PE classes. In addition, we did not use DXA for body composition assessment, which is more accurate for tracking changes in body composition. 

The study was constrained to a ten-week intervention consisting of once-weekly sessions during the school term. These conditions were imposed due to the simultaneous implementation of a standard PE program. Additionally, limitations were identified in the duration of the intervention, which was only 14 min within a 45 min lesson. Considering the need for a warm-up before HIIT and a cooldown afterward, the intervention time was further reduced [11]. On the other hand, it should be noted that HIIT with a longer duration may pose potential risks and increase the likelihood of adverse effects, leading to adolescents’ reluctance to participate in the study.

It is essential to conduct follow-up studies to assess the durability of the observed changes. Measuring CRF in a more objective manner, through maximal oxygen uptake (VO2 max), than can be achieved with the HST is necessary. The step test has inherent limitations and does not provide a precise assessment of CRF. Hence, alternative methods should be considered to accurately evaluate CRF.

## 5. Conclusions

In conclusion, the first hypothesis was confirmed, and the second was partially confirmed. A HIIT effect was detected in body composition, resting BP (both SBP and DBP), CRF, and ABS, from an individual response to the stimulus point of view. These findings confirm the effectiveness of introducing the intervention program as a regular part of a typical PE lesson, even if only conducted once a week. On the other hand, the lack of positive effects in the WHR and most musculoskeletal fitness variables (HS, FLEX, AG, and VJ) suggests the need to continue searching for a more optimal and effective program. The relationship with sex and biological maturation, but not BMI, indicates a need to adapt the program to gender and produce separate programs for males and females. In the future, it is important to conduct studies that assess the long-term sustainability of the effects of the HIIT intervention on weight management and overall health in child and adolescent populations [64]. The current findings have the potential to offer valuable insights to clinicians and healthcare professionals regarding the practical implementation of exercise training programs and regarding indicated modalities for individuals for increased positive response after HIIT. 

## Figures and Tables

**Table 1:** Mean, sd, and 95% (CIs) of changes (Δ) in the experimental group (EG) males and females. The second row contains relative changes (percentages). The results from the *t*-test for independent groups are presented as t-values and *p*-values, and Cohen’s values are given at the end.

Variable	Males				Females				t	*p*	d
	Mean ± SD		95% CI		Mean ± SD		95% CI				
WHR	−0.01	0.03	−0.02	0.00	0.00	0.02	−0.01	0.00	−1.36	0.179	0.311
Δ%	−1.34	3.73	−2.71	0.02	−0.36	2.73	−1.20	0.49	1.31	0.194	0.303
BFP	−1.76	4.45	−3.40	−0.13	−0.15	2.29	−0.87	0.56	−2.01	0.048 *	0.455
Δ%	−8.24	21.69	−16.19	−0.28	−0.46	8.98	−3.26	2.34	2.10	0.040 *	0.469
SMM	0.72	0.75	0.45	1.00	0.34	0.99	0.03	0.65	1.82	0.073	−0.440
Δ%	3.38	3.64	2.04	4.72	1.06	3.40	0.00	2.12	−2.80	0.007 *	−0.659
SBP	−8.19	8.77	−11.41	−4.98	−4.93	8.23	−7.49	−2.36	−1.63	0.108	0.384
Δ%	−6.15	6.32	−8.47	−3.84	−3.94	6.78	−6.05	−1.82	1.42	0.160	0.338
DBP	−1.71	7.46	−4.45	1.03	−2.83	9.14	−5.68	0.01	0.56	0.577	−0.135
Δ%	−1.58	10.22	−5.33	2.17	−3.31	13.31	−7.46	0.83	−0.60	0.548	−0.146
FI	3.01	4.22	1.46	4.56	1.88	4.04	0.62	3.14	1.16	0.250	−0.274
Δ%	7.00	9.22	3.61	10.38	4.91	9.75	1.88	7.95	−0.92	0.360	−0.219
HS	0.10	2.61	−0.86	1.05	0.55	2.27	−0.16	1.25	−0.79	0.434	0.184
Δ%	0.51	5.62	−1.55	2.57	1.86	7.49	−0.47	4.19	0.84	0.402	0.204
ABS	1.29	3.79	−0.10	2.68	3.98	3.33	2.94	5.01	−3.21	0.002 *	0.753
Δ%	5.98	15.04	0.47	11.50	23.89	22.32	16.93	30.84	3.86	0.000 *	0.941
FLEX	1.71	3.08	0.58	2.84	1.60	2.90	0.69	2.50	0.16	0.871	−0.034
Δ%	12.68	38.39	−1.40	26.76	6.71	12.77	2.73	10.68	−0.94	0.349	−0.209
AG	0.17	0.54	−0.02	0.37	0.14	0.57	−0.03	0.32	0.23	0.815	−0.056
Δ%	1.72	5.31	−0.23	3.67	1.41	4.95	−0.13	2.96	−0.25	0.801	−0.060
VJ	−3.01	6.07	−5.24	−0.78	0.18	5.76	−1.61	1.98	−2.29	0.025 *	0.540
Δ%	−4.36	10.99	−8.40	−0.33	1.38	13.90	−2.96	5.71	1.90	0.061	0.458

Footnote: WHR—waist-to-hip ratio, BFP—body fat percentage, SMM—skeletal muscle mass, SBP—systolic blood pressure, DBP—diastolic blood pressure, FI—fitness index, HS—hand strength, ABS—abdomen strength, FLEX—flexibility, AG—agility, VJ—vertical jump, sd—standard deviation, CI—confidence interval, SD_real_—true variability. * Statistically significant *p* < 0.05.

**Table 2:** Prevalence of the NRs and Rs expressed as numbers and percentages. Goodness-of-fit chi-squared test results (χ^2^-values and *p*-values) and Cohen’s ω (effect size).

Variable	NRs N (%)	Rs N (%)	χ^2^	*p*	ω
WHR	34 (46.57%)	39 (53.43%)	0.34	0.558	0.07
BFP	28 (38.35%)	45 (61.65%)	3.93	0.047 *	0.23
SMM	28 (38.35%)	45 (61.65%)	3.93	0.047 *	0.23
SBP	12 (16.43%)	61 (83.57%)	32.89	<0.001 *	0.67
DBP	24 (32.87%)	49 (67.13%)	8.56	0.003 *	0.34
FI	26 (35.61%)	47 (64.39%)	6.04	0.014 *	0.29
HS	49 (67.12%)	24 (32.88%)	8.56	0.003 *	0.34
ABS	26 (35.61%)	47 (64.39%)	6.04	0.014 *	0.29
FLEX	30 (41.09%)	43 (58.91%)	2.32	0.130	0.18
AG	41 (56.16%)	32 (43.84%)	1.11	0.292	0.29
VJ	48 (65.75%)	25 (34.25%)	7.25	0.007 *	0.32

Footnote: NRs—non-responders, Rs—responders, WHR—waist-to-hip ratio, BFP—body fat percentage, SMM—skeletal muscle mass, SBP—systolic blood pressure, DBP—diastolic blood pressure, FI—fitness index, HS—hand strength, ABS—abdomen strength, FLEX—flexibility, AG—agility, VJ—vertical jump, ω—Cohen’s omega. * Statistically significant *p* < 0.05.

**Table 3:** The prevalence of the NRs and Rs, based on sex, expressed by numbers (N) and percentages (%). Chi-squared values, *p*-values, Cramer’s V, and odds ratios (OR).

Variable	Boys				Girls				χ^2^	*p*	Cramer’s V	OR (95% CI)
	NRs N (%)		Rs N (%)		NRs N (%)		Rs N (%)					
WHR	14	45.16%	17	54.84%	20	47.62%	22	52.38%	0.04	0.835	0.02	1.10 (0.04–2.80)
BFP	10	32.26%	21	67.74%	18	42.86%	24	57.14%	0.85	0.357	0.11	1.58 (0.60–4.15)
SMM	8	25.81%	23	74.19%	20	47.62%	22	52.38%	3.59	0.058	0.21	2.61 0.95–7.15
SBP	5	16.13%	26	83.87%	7	16.67%	35	83.33%	0.01	0.951	0.01	1.04 0.29–3.64
DBP	12	38.71%	19	61.29%	12	28.57%	30	71.43%	0.83	0.362	0.11	0.63 0.23–1.69
FI	8	25.81%	23	74.19%	18	42.86%	24	57.14%	2.26	0.132	0.17	2.15 0.78–5.92
HS	23	74.19%	8	25.81%	26	61.90%	16	38.10%	1.22	0.269	0.12	0.56 0.20–1.56
ABS	15	48.39%	16	51.61%	11	26.19%	31	73.81%	3.83	0.050 *	0.22	0.38 0.14–1.01
FLEX	12	38.71%	19	61.29%	18	42.86%	24	57.14%	0.13	0.721	0.04	1.18 0.46–3.05
AG	18	58.06%	13	41.94%	23	54.76%	19	45.24%	0.07	0.779	0.03	0.87 0.34–2.23
VJ	23	74.19%	8	25.81%	25	59.52%	17	40.48%	1.70	0.192	0.15	0.51 0.18–1.41

Footnote: NRs—non-responders, Rs—responders, WHR—waist-to-hip ratio, BFP—body fat percentage, SMM—skeletal muscle mass, SBP—systolic blood pressure, DBP—diastolic blood pressure, FI—fitness index, HS—hand strength, ABS—abdomen strength, FLEX—flexibility, AG—agility, VJ—vertical jump, OR—odds ratio, CI—confidence interval. * Statistically significant *p* < 0.05.

**Table 4:** The prevalence of the NRs and Rs, based on BMI categories, expressed by numbers (N) and percentages (%). Chi-squared values, *p*-values, Cramer’s V, and odds ratios (OR).

Variable	BMI < 20				BMI > 20				χ^2^	*p*	Cramer’s V	OR (95% CI)
	NRs N (%)		Rs N (%)		NRs N (%)		Rs N (%)					
WHR	15	46.88%	17	53.13%	19	46.34%	22	53.66%	0.01	0.964	0.01	1.02 0.40–2.58
BFP	12	37.50%	20	62.50%	16	39.02%	25	60.98%	0.18	0.894	0.02	0.94 0.36–2.43
SMM	12	37.50%	20	62.50%	16	39.02%	25	60.98%	0.18	0.894	0.02	0.94 0.36–2.43
SBP	5	15.63%	27	84.38%	7	17.07%	34	82.93%	0.03	0.868	0.02	0.90 0.26–3.15
DBP	7	21.88%	25	78.13%	17	41.46%	24	58.54%	3.12	0.080	0.20	0.40 0.14–1.12
FI	11	34.38%	21	65.63%	15	36.59%	26	63.41%	0.04	0.845	0.02	0.91 0.35–2.39
HS	20	62.50%	12	37.50%	29	70.73%	12	29.27%	0.55	0.46	0.09	0.69 0.26–1.84
ABS	12	37.50%	20	62.50%	14	34.15%	27	65.85%	0.09	0.767	0.03	1.15 0.44–3.03
FLEX	16	50.00%	16	50.00%	14	34.15%	27	65.85%	1.87	0.172	0.16	1.93 0.75–4.97
AG	18	56.25%	14	43.75%	23	56.10%	18	43.90%	0.01	0.990	0.00	1.01 0.40–2.55
VJ	21	65.63%	11	34.38%	27	65.85%	14	34.15%	0.01	0.984	0.00	0.99 0.37–2.62

Footnote: BMI—body mass index, NRs—non-responders, Rs -responders, WHR—waist-to-hip ratio, BFP—body fat percentage, SMM—skeletal muscle mass, SBP—systolic blood pressure, DBP—diastolic blood pressure, FI—fitness index, HS—hand strength, ABS—abdomen strength, FLEX—flexibility, AG—agility, VJ—vertical jump, OR—odds ratio, CI—confidence interval.

**Table 5:** The prevalence of Rs and NRs in MO categories (biologically younger adolescents (BYA) and biologically advanced adolescents (BAA)), expressed by numbers (N) and percentages (%). Chi-squared values, *p*-values, Cramer’s V, and odds ratios (OR).

Variable	BYA				BAA				χ^2^	*p*	Cramer’s V	OR (95% CI)
	NRs N (%)		Rs N (%)		NRs N (%)		Rs N (%)					
WHR	25	48.08%	27	51.92%	9	42.86%	12	57.14%	0.16	0.686	0.05	1.23 0.44–3.43
BFP	20	38.46%	32	61.54%	8	38.10%	13	61.90%	0.00	0.977	0.00	1.01 0.36–2.88
SMM	16	30.77%	36	69.23%	12	57.14%	9	42.86%	4.40	0.036 *	0.24	0.33 0.12–0.95
SBP	12	23.08%	40	76.92%	0	0.00%	21	100.00%	5.78	0.016 *	0.27	-
DBP	20	38.46%	32	61.54%	4	19.05%	17	80.95%	2.55	0.110	0.18	2.66 0.78–9.03
FI	16	30.77%	36	69.23%	10	47.62%	11	52.38%	1.85	0.174	0.16	0.49 0.17–1.38
HS	36	69.23%	16	30.77%	13	61.90%	8	38.10%	0.36	0.546	0.07	1.38 0.480–3.99
ABS	19	36.54%	33	63.46%	7	33.33%	14	66.67%	0.07	0.796	0.03	1.15 0.40–3.35
FLEX	19	36.54%	33	63.46%	11	52.38%	10	47.62%	1.55	0.213	0.14	0.52 0.19–1.46
AG	31	59.62%	21	40.38%	10	47.62%	11	52.38%	0.87	0.350	0.11	1.62 0.59–4.50
VJ	34	65.38%	18	34.62%	14	66.67%	7	33.33%	0.1	0.920	0.01	0.94 0.32–2.76

Footnote: BYA—biologically younger adolescents, BAA—biologically advanced adolescents, NRs—non-responders, Rs—responders, WHR—waist-to-hip ratio, BFP—body fat percentage, SMM—skeletal muscle mass, SBP—systolic blood pressure, DBP—diastolic blood pressure, FI—fitness index, HS—hand strength, ABS—abdomen strength, FLEX—flexibility, AG—agility, VJ—vertical jump, OR—odds ratio, CI—confidence interval. * Statistically significant *p* < 0.05.

## Data Availability

The data presented in this study are available upon request from the corresponding author.
